# Effector specificity and function in Drosophila innate immunity: Getting AMPed and dropping Boms

**DOI:** 10.1371/journal.ppat.1008480

**Published:** 2020-05-28

**Authors:** Samuel J. H. Lin, Lianne B. Cohen, Steven A. Wasserman

**Affiliations:** Section of Cell and Developmental Biology, Division of Biological Sciences, University of California, San Diego, La Jolla, California, United States of America; University of Massachusetts, Worcester, UNITED STATES

Our understanding of *Drosophila* innate immunity has seen major advances in the last five years, catalyzed by two transformative technologies—genome editing and genome-wide association studies—as well as by insights gained from the parallel study of pathogen growth and host survival following infection. As a result, researchers have characterized novel and essential effectors, rewritten the individual and collective roles of antimicrobial peptides, and identified stochastic variation and persistent infection as common features of microbial infection. We focus here on the inducible cell-free response of *Drosophila melanogaster* to bacterial or fungal pathogens. Readers interested in innate immunity in other insects, defenses in the gut and other organs, cellular immunity, or antiviral mechanisms are encouraged to consult recent reviews on these topics [1–6].

## Essential roles for novel peptide effectors

Upon infection in hosts ranging from flies to humans, pathogen associated molecular patterns (PAMPs) initiate nonself recognition that triggers conserved innate immune signaling pathways [7]. In *Drosophila*, the Toll pathway responds to fungi and most gram-positive bacteria, whereas the Imd pathway responds to gram-negative bacteria and those gram-positive bacteria with a similar peptidoglycan structure [8]. Each pathway induces the synthesis and secretion of effector molecules that circulate in the hemolymph and combat bacterial and fungal invaders. The best-known effectors are the antimicrobial peptides (AMPs), which can be viewed as ribosomally synthesized antibiotics [9,10].

Until recently, little was known about the contributions of particular peptides to in vivo immune defenses. The paucity of knowledge reflected the near absence of loss-of-function mutations, a scarcity with several likely causes. First, peptide genes offer small targets for random mutagenesis. Second, many belong to gene families, potentially minimizing the loss-of-function phenotype for an individual gene mutation. Third, immune mutant screens have typically assayed for loss of reporter gene activation and thus identified lesions only in genes upstream of effector induction [see, e.g., 11,12].

**Fig 1:**
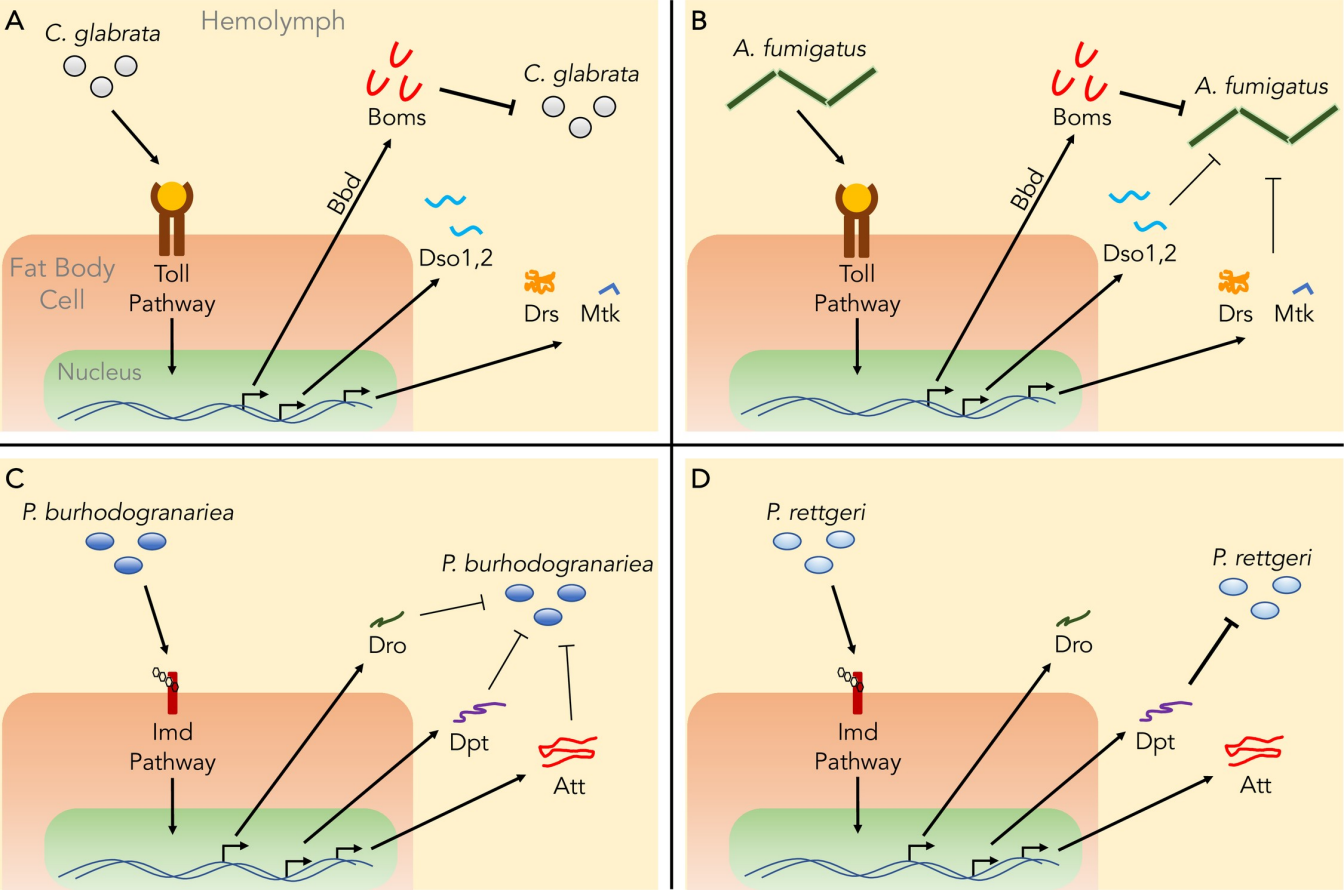
Specificity of *Drosophila* immune effectors. A–D each depict effectors’ contribution to defense against a particular pathogen, as deduced from loss-of-function phenotypes. Thick lines represent strong phenotypes, and thin lines represent weaker phenotypes. The effector repertoires induced by Toll (A and B) and by Imd (C and D) are largely invariant, but the subset of effectors that mediate the response to a given challenge varies. (A) The Boms and Bbd are required for survival against the yeast *C*. *glabrata* [13,15,16]. (B) Boms, Bbd, Dso1,2, Drs, and Mtk are necessary for survival against the filamentous fungus *Aspergillus fumigatus* [14,16,17]. (C) A number of AMPs (Dro, Dpt, and Att) overlap in function in providing defense against the gram-negative bacterium *Providencia burhodogranariea* [14]. (D) Dpt is the sole essential mediator of defense against the gram-negative bacterium *P*. *rettgeri* [14,24]. AMP, antimicrobial peptide; Att, Attacin; Bbd, Bombardier; Boms, Bomanins; Dso1,2, Daisho1 and Daisho2; Dpt, Diptericin; Dro, Drosocin; Drs, Drosomycin; Mtk, Metchnikowin.

The advent of genome editing technologies reinvigorated the genetic study of innate immunity. One breakthrough came with the deletion of a gene cluster encoding members of a peptide family now known as the Bomanins (or Boms) [13]. The Bom peptides lack sequence similarity to prototypical AMPs or, indeed, any proteins of known structure or function. Remarkably, excising 10 of the 12 *Bom* genes disrupts immune defenses against a range of pathogens to the same extent and with the same specificity as blocking Toll signaling (Fig 1A and Fig 1B) [13,14]. In the case of the pathogen *Candida glabrata*, a yeast, Boms are essential not only for survival of infected flies but also for humoral candidacidal activity [15].

Recent experiments have demonstrated immune functions for additional Toll-induced effectors. One such study demonstrated that the hemolymph of flies lacking the Bombardier protein specifically fails to accumulate the short-form Bom peptides [16]. Furthermore, disrupting the *bombardier* gene reduces survival against the same set of pathogens as does deleting the *Bom* gene cluster, consistent with a prior study indicating a primary role in defense for the short-form Boms [15]. There are also Toll-induced effectors essential for a subset of Toll-mediated defenses. For example, the peptides encoded by the two *daisho* genes mediate survival against a subset of filamentous fungi, such as *A*. *fumigatus* (Fig 1B) [17].

## Differential requirement for Drosophila AMP function

From 1980 onward, the study of innate immune effectors largely centered on AMPs. Each of the seven prototypical *Drosophila* AMPs has potent in vitro activity against a subset of pathogens. Drosomycin, for example, is active against *Neurospora crassa* and certain other filamentous fungi at concentrations of 1 μM or lower [18]. Furthermore, constitutive expression of Drosomycin in vivo in the absence of Toll or Imd activation enables survival of flies infected with *N*. *crassa* [19].

In 2019, a CRISPR/Cas9-based study directly addressed the requirement for the prototypical AMP genes in innate immune defense [14]. The underlying idea was simple and elegant—to knockout AMP genes singly, in sets, and in toto and then examine the phenotypic consequences upon infection with various pathogens. In the case of the Imd pathway, flies simultaneously deleted for the majority of induced AMP genes are as susceptible to gram-negative bacteria as those lacking all pathway function [14]. Moreover, for many gram-negative bacteria, individual gene disruption is without consequence, supporting the idea that organisms express sets of AMPs that overlap in specificity and function (Fig 1C). In the case of the Toll pathway, however, deleting most AMP genes decreases resistance markedly against only a subset of fungi and has little or no effect on survival upon infection with gram-positive bacteria.

Taken together, the loss-of-function phenotypes for the Bomanins and the prototypical AMPs indicate that Drosomycin induced upon infection is neither strictly required nor sufficient for defense against a filamentous fungus. Yet when constitutively expressed, Drosomycin enables survival of immune deficient flies infected with a filamentous fungus [19]. How can this paradox be resolved? One idea, offered in the context of another insect host (*Tenebrio molitor*), is that induced peptides accumulate too long after infection to be primary effectors [20]. In general, this hypothesis is a poor fit in *Drosophila*, since induced AMPs and Bomanins are strictly essential for defenses mediated by Imd and Toll, respectively. It may, however, accurately describe the limited effectiveness of the Toll-induced expression of prototypical AMPs in the most common experimental model for *Drosophila*—a sudden and massive systemic infection introduced by injecting or stabbing thousands of pathogens into the fly body.

## Evolutionary selection for *Drosophila* AMP function

From 2000 to 2010, association studies using infection with gram-negative bacteria failed to identify SNPs in AMPs for which the SNP state was predictive of susceptibility [21–23]. Instead, signatures of selection were largely confined to factors mediating pathogen recognition or signaling transduction. The interpretation at the time was that functional redundancy among the AMPs was sufficient to preclude a mutation in any individual AMP gene from having a significant effect on resistance.

Although AMP redundancy is pervasive, it is not universal. In 2016, SNPs were identified in the gene for the AMP Diptericin that are associated with higher pathogen load after infection with the gram-negative bacterium *P*. *rettgeri* [24]. Flies null for *Diptericin* sustain higher loads with *P*. *rettgeri* than do the wild type and succumb rapidly after infection [14]. It thus appears that Diptericin is the sole mediator of defense against *P*. *rettgeri* (Fig 1D). At the same time, *Diptericin* appears to be functionally redundant or irrelevant against other pathogens, as variation at the locus has no detectable effect upon infection with a range of other gram-negative bacteria, including some members of the *Providencia* genus [24]. Finally, we note that Diptericin null alleles turn out to be common in Africa, consistent with a link between AMP sequence evolution and variation in ecological niche [25].

## A new model for the outcome of innate immunity

Innate immune effector function in vivo is often assayed by postinfection host survival, which was long assumed to reflect pathogen clearance. One of the first contraindications came in 2013, when it was found that wild-type flies survive infection with *C*. *glabrata* but do so without clearing the pathogen [26]. Because humans also fail to clear *C*. *glabrata* infections [27], it was suggested that persistent infection of flies reflected a particular property of this yeast rather than any general feature of innate immunity in the host. In 2017, however, a study revealed that flies surviving infection with any of a range of bacteria also remain infected indefinitely [28]. Furthermore, this holds true for both gram-negative and gram-positive bacteria, and thus is not linked to whether or not AMPs are the primary immune effectors.

The 2017 report on stochastic variation described two stereotypic outcomes for infection of individual flies matched for age, genotype, environment, and infection: Either the pathogen replicates, reaches a lethal burden, and the fly dies; or the immune system controls the pathogen at a load below the lethal burden, resulting in survival accompanied by persistent low-level infection. The variation observed in survival curves among these flies reflects stochasticity in the time required to reach lethal burden and in the fraction of flies that control the infection before it reaches that threshold. The practical impact of this model is a recognition of the importance of pathogen load upon death as a powerful tool both for comparing innate responses to infection and for determining whether a particular genotype affects tolerance to infection [29].

## Open questions and future directions

How do the novel Toll effectors provide protection? To date, the delineation of molecular mechanisms in innate immune pathways has benefited considerably from the synergy of exploring highly conserved pathogen recognition and signaling molecules in evolutionarily diverged contexts. A similar approach will not be possible for the *Drosophila* effectors: the Bomanins and the Daisho peptides are all restricted to *Drosophila* and *Scaptodrosophila* [13,17,25]. They are not unique in that aspect, as taxonomically restricted genes (TRGs) form a major fraction of the induced immune repertoire in many species [30–32]. Nevertheless, the absence of known structure or activity for these TRGs provides a significant challenge.

A second major question going forward is how infection persists in the presence of effectors that block pathogen growth and accumulation. Are some viable pathogens sequestered in a clot or other extracellular structure? Alternatively, are a fraction of pathogens in systemic infection engulfed by phagocytic hemocytes, but not killed?

Finally, much remains uncertain regarding selective pressures on immune effectors. To what extent do differences in AMP sequence contribute to defense against infection? Relevant findings have been reported not only for Diptericin, as discussed above, but also for other prototypical AMPs, including Drosocin [14]. In addressing this topic, and immune system function and evolution more generally, it will be important to consider to what extent the “shock and awe” of infection with a megadose of a single pathogen recapitulates the typical challenges in the life of a fruit fly.
